# Feasibility of four-dimensional similarity filter for radiation dose reduction in dynamic myocardial computed tomography perfusion imaging

**DOI:** 10.3389/fradi.2023.1214521

**Published:** 2023-12-01

**Authors:** Yuta Yamamoto, Yuki Tanabe, Akira Kurata, Shuhei Yamamoto, Tomoyuki Kido, Teruyoshi Uetani, Shuntaro Ikeda, Shota Nakano, Osamu Yamaguchi, Teruhito Kido

**Affiliations:** ^1^ Department of Radiology, Ehime University Graduate School of Medicine, Toon, Japan; ^2^ Department of Cardiology, National Hospital Organization Shikoku Cancer Center, Matsuyama, Japan; ^3^ Department of Cardiology, Pulmonology, Hypertension and Nephrology, Ehime University Graduate School of Medicine, Toon, Japan; ^4^ Canon Medical Systems Corporation, Otawara, Japan

**Keywords:** computed tomography perfusion, four-dimensional similarity filter, radiation dose reduction, signal-to-noise ratio, myocardial blood flow

## Abstract

**Rationale and objectives:**

We aimed to evaluate the impact of four-dimensional noise reduction filtering using a four-dimensional similarity filter (4D-SF) on radiation dose reduction in dynamic myocardial computed tomography perfusion (CTP).

**Materials and methods:**

Forty-three patients who underwent dynamic myocardial CTP using 320-row computed tomography (CT) were included in the study. The original images were reconstructed using iterative reconstruction (IR). Three different CTP datasets with simulated noise, corresponding to 25%, 50%, and 75% reduction of the original dose (300 mA), were reconstructed using a combination of IR and 4D-SF. The signal-to-noise ratio (SNR) and contrast-to-noise ratio (CNR) were assessed, and CT-derived myocardial blood flow (CT-MBF) was quantified. The results were compared between the original and simulated images with radiation dose reduction.

**Results:**

The median SNR (first quartile–third quartile) at the original, 25%-, 50%-, and 75%-dose reduced-simulated images with 4D-SF was 8.3 (6.5–10.2), 16.5 (11.9–21.7), 15.6 (11.0–20.1), and 12.8 (8.8–18.1) and that of CNR was 4.4 (3.2–5.8), 6.7 (4.6–10.3), 6.6 (4.3–10.1), and 5.5 (3.5–9.1), respectively. All the dose-reduced-simulated CTPs with 4D-SF had significantly higher image quality scores in SNR and CNR than the original ones (25%-, 50%-, and 75%-dose reduced vs. original images, *p* < 0.05, in each). The CT-MBF in 75%-dose reduced-simulated CTP was significantly lower than 25%-, 50%- dose-reduced-simulated, and original CTPs (vs. 75%-dose reduced-simulated images, *p* < 0.05, in each).

**Conclusion:**

4D-SF has the potential to reduce the radiation dose associated with dynamic myocardial CTP imaging by half, without impairing the robustness of MBF quantification.

## 1. Introduction

Assessment of myocardial ischemia is crucial for the management of coronary artery disease (CAD) (1, 2). Coronary computed tomography angiography (CTA) is widely used for the assessment of coronary artery stenosis. However, CTA-based stenosis severity assessment still has some limitations in identifying hemodynamically significant CAD (3). Myocardial computed tomography perfusion (CTP) imaging has emerged as a useful tool for myocardial perfusion imaging; dynamic CTP imaging allows for quantitative assessment of myocardial perfusion by quantifying hemodynamic parameters such as myocardial blood flow (MBF) (4, 5). However, high radiation doses are inevitable with dynamic myocardial CTP imaging, because multiple scans are required during the first pass of contrast medium in the myocardium. The balance between the radiation dose and image quality should be considered in clinical practice (6).

Iterative reconstruction (IR) is an effective method for reducing image noise and radiation dose by combining the low-tube current scan (7, 8). However, IR lacks temporal regularization in the time attenuation curve and impairs spatial resolution if the radiation dose is reduced excessively (9). Therefore, other specific techniques are required for reducing the radiation dose associated with dynamic myocardial CTP imaging while maintaining diagnostic image quality. A recent study has shown that a four-dimensional noise reduction filter using a similarity algorithm [four-dimensional similarity filter (4D-SF)] could be complementarily used with IR to improve the image quality without altering the CT-MBF values (10). Previous studies have investigated the image quality of dynamic myocardial CTP imaging using spatiotemporal noise reduction techniques (10–12). However, no study assessed the influence of a spatiotemporal noise reduction filter utilized for radiation dose reduction in the CT-MBF quantification. This study aimed to investigate the possibility of radiation dose reduction and CT-MBF robustness in dynamic myocardial CTP with 4D-SF.

## 2. Materials and methods

### 2.1. Study population

The institutional ethics committee of Ehime University Hospital approved this retrospective observational study (registration number: 1910006) and waived the need for informed consent. We identified from the clinical database 50 patients who underwent stress dynamic CTP scanning for the assessment of CAD at the attending physician's discretion between September 2017 and September 2019. We excluded patients with (1) low left ventricular ejection fraction < 20%, (2) arrhythmia, (3) greater than first-degree atrioventricular block, or (4) inappropriate CTP data for CT-derived MBF quantification. Coronary artery stenosis ≥50% on CTA was considered significant and was classified based on the three major coronary vessels; patients were assessed on a per-vessel basis. The effective radiation dose was calculated from the dose-length product in a dose report (conversion factor = 0.014) (13).

### 2.2. Scan protocol of stress dynamic CTP

Stress dynamic CTP was performed using a 320-row multi-detector CT system (Aquilion ONE GENESIS Edition; Canon Medical Systems, Otawara, Japan) as a part of the comprehensive cardiac CT protocol with a partial modification of the previous protocol (10). The scan timing of dynamic CTP was independently optimized, with the timing bolus scan using a 20%-diluted contrast medium set at 6 s before the arrival of the contrast medium at the ascending aorta. Contrast medium (iopamidol, 370 mg iodine/ml; Bayer Yakuhin, Osaka, Japan) and a saline chaser were administered at the same injection rate and volume as the timing bolus scan, 3 min after adenosine triphosphate loading (0.16 mg/kg/min). The stress dynamic CTP dataset was obtained using the prospective electrocardiogram-gated dynamic mode, targeting a phase of 45% of the RR interval. The scan parameters for CTP were as follows: tube voltage, 80 kVp; tube current, 300 mA; gantry rotation speed, 0.275 s/rotation; detector collimation, 320 × 0.50 mm; and effective coverage, 100 mm. Subsequently, coronary CTA and delayed-enhancement CT were performed at 10 min and 15 min after stress dynamic CTP, respectively.

### 2.3. Post-processing of dynamic myocardial CTP images

**Figure 1:**
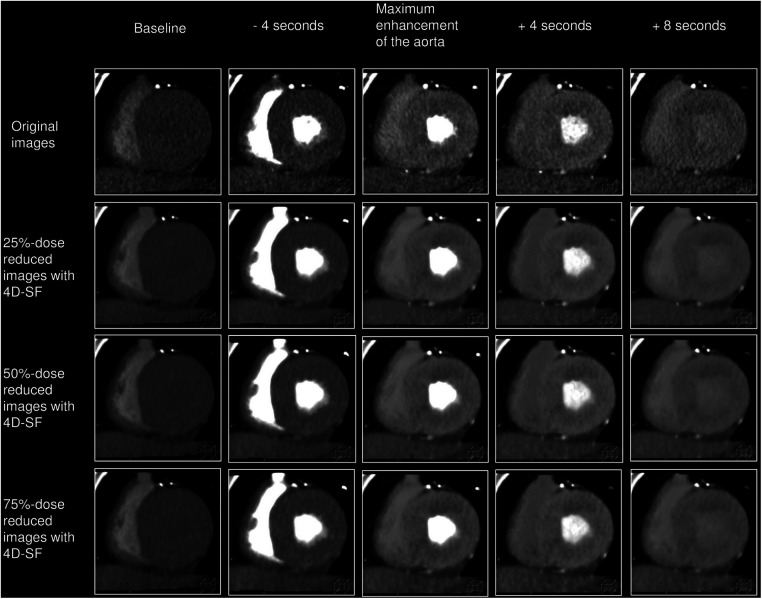
Post-processing of dynamic myocardial CTP images. Dynamic CTP images were reconstructed with AIDR 3D and the non-rigid registration algorithm, as the original images. Three other CTP datasets were generated by adding simulated noise corresponding to the dose reduction rate (25%-, 50%-, and 75%-dose reduced-simulated images). The image filtering process with 4D-SF was used after noise simulation processing. 4D-SF, four-dimensional similarity filter; AIDR 3D, adaptive iterative dose reduction in three dimensions; CTP, computed tomography perfusion.

A 360° full-reconstruction algorithm, adaptive iterative dose reduction in three-dimensional processing (AIDR 3D, FC03, strong), and non-rigid registration algorithm for motion compensation were used for CTP image reconstruction. The trans-axial images in the dynamic CTP dataset were reconstructed with 1.0-mm slice thickness, as the original images. In addition, three other CTP datasets were generated by adding simulated noise corresponding to the dose reduction rate (presented as tube amplitude for the dose) through a statistical model tool including both quantum noise and electronic noise (14). These datasets had values with 25% (225 mA), 50% (150 mA), and 75% dose reductions (75 mA) from the original dose (300 mA). The 4D-SF was used by specific software in the commercially available workstation (Vitrea; Canon Medical Systems). The image filtering process with 4D-SF was performed after noise simulation processing (Figure 1). Finally, the four CTP datasets (original, 25%-, 50%-, and 75%-dose reduced-simulated images with 4D-SF) were evaluated using a dedicated workstation (Vitrea, Canon Medical Systems) for image quality and CT-MBF analyses.

### 2.4. Image quality analyses

Image quality analyses were performed for the four different CTP datasets using 5-mm-thick cardiac short-axis CTP images by average intensity projection reformat. An experienced radiologist (with 6 years of experience in cardiac imaging) selected a representative single phase (approximately 4 s after the time point of maximal enhancement in the ascending aorta) at the optimal phase for the assessment of myocardial ischemia from a series of dynamic CTP datasets, as previously described (15).

Regarding qualitative image parameters, two radiologists (with 5 and 6 years of experience in cardiac imaging), who were blinded to all clinical and reconstruction information, independently evaluated the four different CTP datasets in random order in terms of the noise, contrast, and contour sharpness using a 5-grade scale (1, non-diagnostic; 2, fair; 3, moderate; 4, good; and 5, excellent) in the optimal window level/width settings for each case (16). Discrepancies between the two observers were resolved by consensus.

Regarding quantitative image parameters, the fourth experienced radiologist (with 6 years of experience in cardiac imaging) evaluated myocardial CT attenuation (Hounsfield unit) and the standard deviation (SD) by placing regions of interest (ROIs) (100–150 mm^2^) in the center of each myocardial segment based on a 16-segment model without the apex (17). The SD was defined as image noise. The ROI (50–100 mm^2^) in the nearby skeletal muscle (latissimus dorsi, pectoralis, or intercostal) was defined as the reference tissue (16). The signal-to-noise ratio (SNR) was calculated by dividing the myocardial CT attenuation of an ROI by the SD of the same ROI. The contrast-to-noise ratio (CNR) was calculated by dividing the difference in CT attenuation between the myocardium and reference tissue by the SD of the reference tissue (16).

### 2.5. CT-derived myocardial blood flow analyses

Two experienced radiologists (with 5 and 6 years of experience in cardiac imaging) independently analyzed myocardial peak CT attenuation, time to peak (TTP), and CT-MBF in the four different dynamic CTP datasets. CT-MBF was semi-automatically quantified using the Renkin-Crone equation, validated with ^15^O-labelled water positron emission tomography (18). Global CT-MBF was defined as the mean of all 16 segmental values.

### 2.6. Statistical analyses

Continuous data were expressed as the mean (SD) or as the median (first quartile–third quartile) according to the distribution. Regarding the intra- and inter-observer agreements, Cohen kappa (k) statistics were used for the visual image quality scores and intra-class correlation coefficients (ICC) were used for the quantitative CTP-derived quantitative parameters such as peak CT attenuation, TTP, and CT-MBF. Differences were compared between the original images and each simulated CTP image using the Dunnett test or the Steel-Dwass test. In all tests, statistical significance was determined at *p* < 0.05. Statistical analyses were performed using JMP13 (SAS Institute, Cary, NC).

## 3. Results

### 3.1. Study population

Among the 50 patients who underwent stress dynamic myocardial CTP, 7 were excluded because of inappropriate CTP datasets due to insufficient breath-holding during the scan (*n* = 4) or beam-hardening artifacts (*n* = 3). Finally, 43 eligible patients were enrolled in the present study (Table 1). The median estimated radiation dose was 3.8 (3.3–4.2) mSv for the original dynamic myocardial CTP data acquisition.

**Table 1:** Patient characteristics.

Age (years)	68.4 (7.4)
Male (% of total)	32 (74%)
Body mass index (kg/m^2^)	24.8 (3.3)
Coronary risk factors (*n*, %)	
Hypertension	29 (67%)
Dyslipidemia	21 (49%)
Diabetes mellitus	20 (47%)
Smoking habit	27 (63%)
Family history of CAD	12 (28%)
Chest pain (*n*, %)	29 (67%)

Data are expressed as the mean (standard derivation), or *N* (%). CAD, coronary artery disease.

### 3.2. Qualitative and quantitative image quality

The image quality results are shown in Table 2. Regarding the score of qualitative image parameters, the kappa values of intra- and inter-observer agreements were 0.92 and 0.88 for noise, 0.95 and 0.92 for contrast, and 0.86 and 0.91 for sharpness, respectively, indicating satisfactory reproducibility (*κ* > 0.70). Each of the dose reduced-simulated CTP images with 4D-SF had a significantly higher visual image quality score in terms of noise than the original images (*p* < 0.001). In contrast, scores of the 75%-dose reduced-simulated CTP images with 4D-SF were significantly lower than those of the 50%-dose reduced-simulated CTP images (*p* < 0.001). No significant difference was observed in contrast and sharpness between the original and the four different simulated CTP images (*p* = 0.057 for contrast, *p* = 0.134 for sharpness).

**Table 2:** Scoring of qualitative and quantitative image parameters in dynamic myocardial CTP with 4D-SF.

	Qualitative image quality			Quantitative image quality		
	Noise	Contrast	Sharpness	SNR	CNR	Image noise
Original image	3.63 (0.49)	4.74 (0.44)	4.70 (0.51)	8.3 (6.5–10.2)	4.4 (3.2–5.8)	14.2 (12.1–17.3)
25%-dose reduction + 4D-SF	4.98 (0.15)*	4.86 (0.35)	4.65 (0.53)	16.5 (11.9–21.7)*	6.7 (4.6–10.3)*	7.2 (5.7–9.5)*
50%-dose reduction + 4D-SF	4.98 (0.15)*	4.84 (0.37)	4.60 (0.54)	15.6 (11.0–20.8)*	6.6 (4.3–10.1)*	7.7 (6.1–9.9)*^#^
75%-dose reduction + 4D-SF	4.56 (0.55)*^†^	4.58 (0.63)	4.56 (0.55)	12.8 (8.8–18.1)*^†^	5.5 (3.5–9.1)*^†^	8.8 (6.9–11.7)*^†^

Data are expressed as the mean (standard deviation) or median (interquartile range). CTP, computed tomography perfusion; 4D-SF, four-dimensional similarity filter; SNR, signal-to-noise ratio; CNR, contrast-to-noise ratio; dose reduction + 4D-SF, dose reduced-simulated image with 4D-SF.

* *p* < 0.001, vs. original CTP images.

^†^ *p* < 0.001, with 75%- vs. 50%-dose reduced-simulated CTP image with 4D-SF.

^#^ *p* < 0.05, vs. 25%-dose reduced-simulated CTP image with 4D-SF.

**Figure 2:**
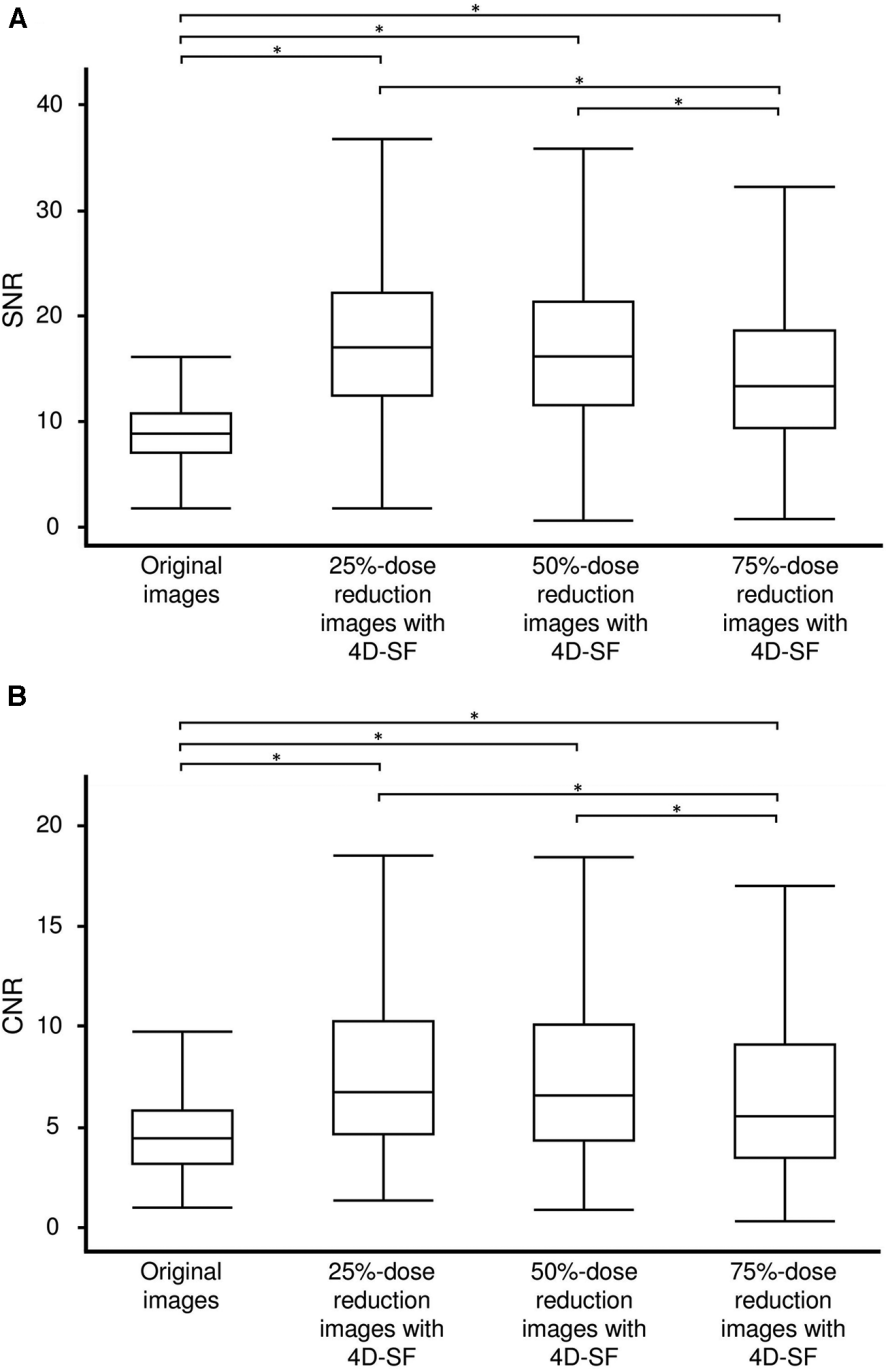
SNR (**A**) and CNR (**B**) in original CTP and 25%-, 50%, and 75%-dose reduced-simulated images with 4D-SF. 4D-SF results in a significant improvement in the SNR and CNR of myocardial CTP images despite dose reduction. Both SNR and CNR were significantly lower in 75%-dose reduced-simulated images with 4D-SF than in 25%- and 50%-dose reduced-simulated images with 4D-SF. 4D-SF, four-dimensional similarity filter; CNR, contrast-to-noise ratio; CTP, computed tomography perfusion; SNR, signal-to-noise ratio. *p < 0.001.

Regarding the score of quantitative image parameters, the median image noise at the original, 25%-, 50%-, and 75%-dose reduced-simulated images with 4D-SF was 14.2 (12.1–17.3), 7.2 (5.7–9.5), 7.7 (6.1–9.1), and 8.8 (6.9–11.7), respectively. While the noise simulation process showed a significant increase in image noise in the order of 25%-, 50%-, and 75%-dose reduced-simulated image (*p* < 0.05, in each), 4D-SF significantly reduced the image noise at each dose reduced-simulated image in comparison with the original image (*p* < 0.001, in each). All the 4D-SF post-processed and dose reduced-simulated CTP images had significantly higher image quality scores in SNR and CNR than the original CTP images (*p* < 0.001, Figure 2). In addition, the 4D-SF post-processed and 75%-dose reduced-simulated CTP images had significantly lower SNR and CNR than the 4D-SF post-processed and 25%- or 50%-dose reduced-simulated CTP images (*p* < 0.001 in each).

### 3.3. CTP-derived quantitative parameters

The results of the CTP-derived quantitative parameters are shown in Table 3. The myocardial peak CT attenuation and TTP were assessed using 10 randomly selected patients (160 myocardial segments) from the study population. The ICCs were 0.95 for myocardial peak CT attenuation and 0.85 for TTP.

**Table 3:** Myocardial peak CT attenuation, TTP, and CT-MBF in dynamic myocardial CTP with 4D-SF.

	Peak CT attenuation (HU)	TTP (s)	CT-MBF (ml/g/min)
Original image	120.5 (16.5)	17.7 (2.9)	2.10 (1.41–2.80)
25%-dose reduction + 4D-SF	119.5 (17.1)	18.0 (3.1)	2.08 (1.41–2.76)
50%-dose reduction + 4D-SF	118.7 (18.0)	18.0 (3.0)	2.07 (1.36–2.83)
75%-dose reduction + 4D-SF	113.3 (22.6)*	17.8 (3.1)	1.82 (0.98–2.64)*

Data are expressed as the mean (standard deviation). CT, computed tomography; TTP, time to peak; CT-MBF, computed tomography-derived myocardial blood flow; CTP, computed tomography perfusion; 4D-SF, four-dimensional similarity filter; HU, hounsfield unit; CNR, contrast-to-noise ratio; HU, hounsfield unit; dose reduction + 4D-SF, dose reduced-simulated image with 4D-SF.

* *p* < 0.05 vs. original CTP images.

The myocardial peak CT attenuation in 75%-dose reduced-simulated images with 4D-SF was significantly lower than that in the original images (*p* < 0.001). However, those in 25%- or 50%-dose reduced-simulated images with 4D-SF were not significantly different from those in the original images (*p *= 0.829 and 0.595). No significant difference was observed in the TTP between the original images and each dose reduced-simulated image with 4D-SF (*p *= 0.431, 0.6750, and 0.873).

**Figure 3:**
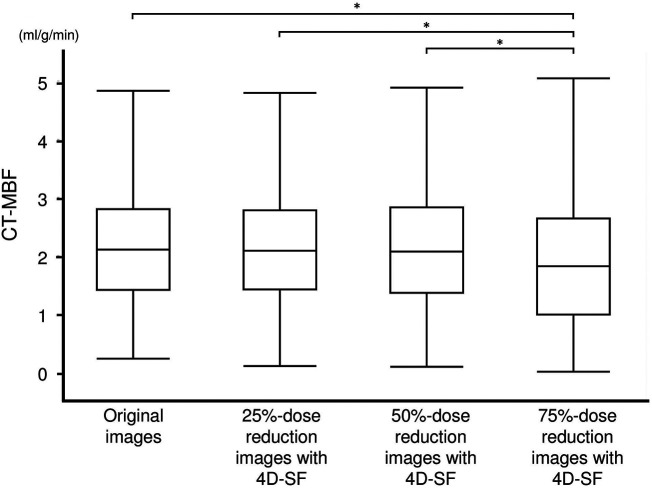
CT-MBF in original CTP and 25%-, 50%, and 75%-dose reduced-simulated images with 4D-SF. 4D-SF did not alter the quantification of CT-MBF in the simulated CTP images with 25% and 50% dose reduction. The CT-MBF in 75%-dose reduced-simulated images with 4D-SF was significantly lower than that in the original and 25%- and 50%-dose reduced-simulated images with 4D-SF. 4D-SF, four-dimensional similarity filter; CT-MBF, computed tomography-derived myocardial blood flow; CTP, computed tomography perfusion. *p < 0.05.

**Figure 4:**
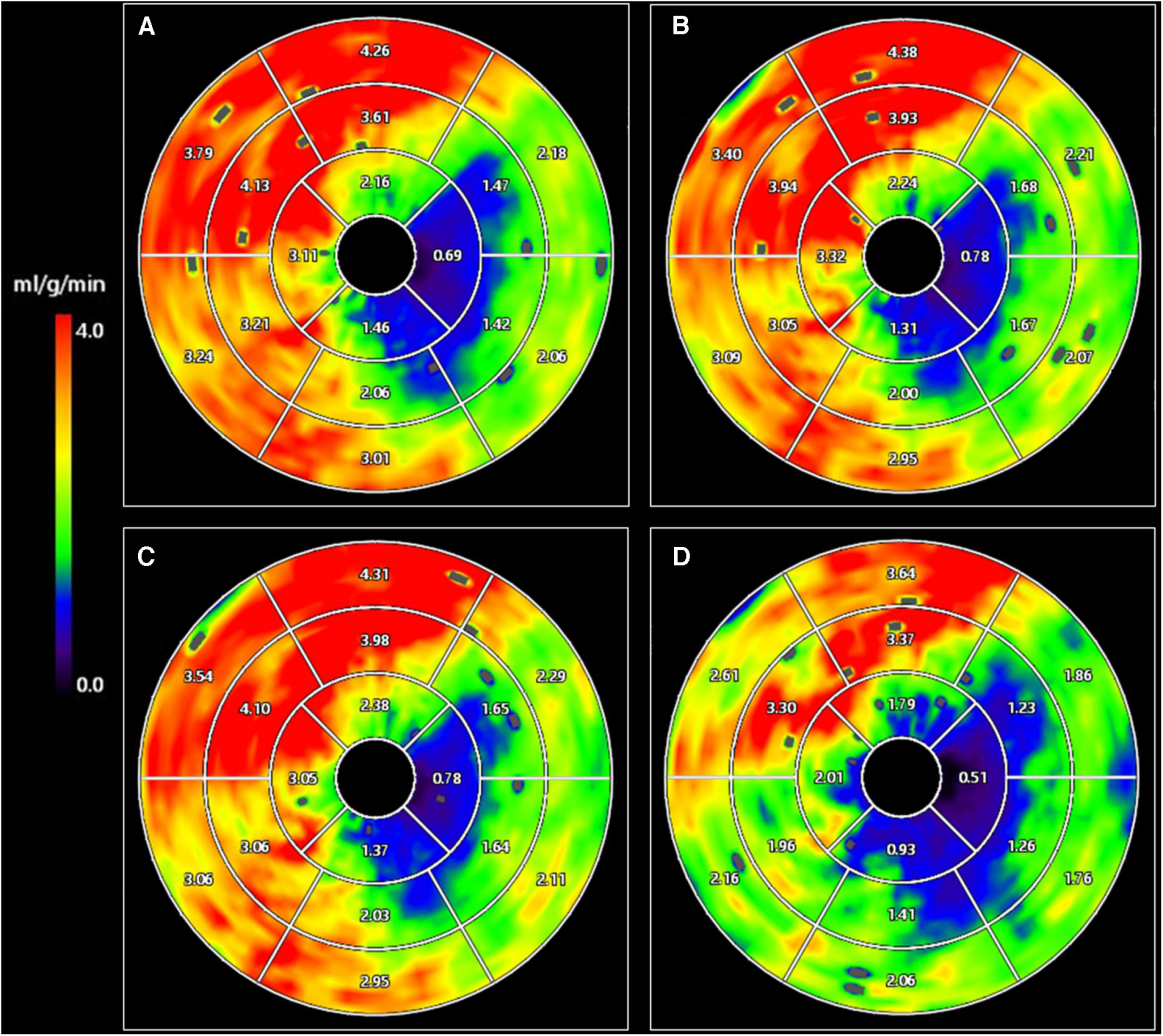
CT-MBF in original CTP images (**A**) and 25%- (**B**), 50%- (**C**), and 75%- (**D**) dose reduced-simulated images with 4D-SF. A 73-year-old man with severe stenosis in the left circumflex coronary artery territory. The mean value of CT-MBF in 75%-dose reduced-simulated images with 4D-SF (1.91 ml/g/min) was lower than that in the original and 25%- and 50%-dose reduced-simulated images (2.53, 2.53, and 2.56 ml/g/min, respectively). 4D-SF, four-dimensional similarity filter; CT-MBF, computed tomography-derived myocardial blood flow; CTP, computed tomography perfusion.

The ICC for CT-MBF assessed using 10 randomly selected patients (160 myocardial segments) was 0.89. The CT-MBF in the original images was 2.10 (1.41–2.80) ml/g/min; those in 25%-, 50%-, and 75%-dose reduced-simulated images with 4D-SF were 2.08 (1.41–2.76), 2.07 (1.36–2.83), and 1.82 (0.98–2.64) ml/g/min, respectively (Figure 3). The CT-MBF in 75%-dose reduced-simulated images with 4D-SF was significantly lower than that in the original images (*p* < 0.001). However, the CT-MBF in 25%- or 50%-dose reduced-simulated images with 4D-SF were not significantly different from those in the original images (*p *= 0.994 and 0.993). A representative case is shown in Figure 4.

## 4. Discussion

In the present study, we showed that (1) 4D-SF could significantly improve qualitative and quantitative image parameters in different dose-simulated CTP images, and (2) 4D-SF did not alter the CT-MBF values in 25%- and 50%-dose reduced-simulated CTP images compared to the original ones.

Dynamic myocardial CTP imaging can have a high diagnostic performance providing an incremental value to CTA for detecting hemodynamically significant CAD assessed with invasive fractional flow reserve (FFR) (19, 20). A recent prospective study by Li et al. has also shown that CTP outperformed CT-FFR in identifying hemodynamically significant CAD (21). However, in dynamic CTP, a relatively high radiation dose remains a concern in the combined use of coronary CTA in clinical practice.

Low-tube voltage scanning is an effective radiation dose reduction method and improves myocardial ischemia detectability by increasing the image contrast between normal and ischemic myocardium. Still, it increases image noise to impair diagnostic image quality (22). Iterative reconstruction reduces image noise and allows lower-tube voltage scans that lead to further radiation dose reduction without impairing image quality (23). However, IR has a limitation in radiation dose reduction because of an over-smoothing effect associated with the left shift in spatial frequency curves toward lower frequencies if the radiation dose is reduced excessively (24). A temporally undersampled scan is also useful for reducing the radiation dose in dynamic myocardial CTP imaging, although undersampling may impair the robustness of CT-MBF quantification (25).

4D-SF is a novel technique that can be combined with IR; it allows for image quality improvement without impairing the robustness of CT-MBF quantification (10). 4D-SF is specifically for the post-processing of dynamic CTP data operating in the temporal domain; the spatial resolution is maintained because 4D-SF does not apply any spatial filtering using neighborhood voxels. Thus, we applied 4D-SF after a non-rigid registration algorithm for motion compensation in CTP image reconstruction. In the present study, 4D-SF has shown improved the qualitative and quantitative image quality in different dose reduced-simulated CTP images. However, the image quality (SNR and CNR) of myocardial CT attenuation in 75%-dose reduced-simulated images was significantly altered in comparison with the original images and resulted in the impairment of robust CT-MBF quantification, despite using 4D-SF.

In the present study, the radiation dose in dynamic myocardial CTP [3.8 (3.3–4.2) mSv]) was lower than that of previous studies (12, 19). It is desirable to reduce the radiation dose as much as possible without impairing diagnostic image quality. The present results indicate that 50%-radiation dose reduced-simulated images with 4D-SF are feasible for dynamic myocardial CTP imaging, and the radiation dose can be reduced to approximately 2 mSv, which is one-quarter of the mean radiation dose (9.45 mSv) used in previous studies (19). 4D-SF has the potential to allow for dynamic myocardial CTP imaging with an extremely low radiation dose (<3 mSv) by combining low tube voltage, low tube current, and undersampled acquisition (25, 26). Moreover, the post-processing of 4D-SF can be applied to dynamic myocardial CTP images in only 2 min using commercial software, substantially improving its availability in clinical practice (10).

This study has several limitations. First, it was a retrospective single-center study with a small sample size. Second, this study did not analyze low-dose dynamic myocardial CTP acquisition data that required comparing the original one because of the risk of multiple radiation exposures. Third, we did not evaluate the combination of 4D-SF with other IR techniques since a single IR method (AIDR 3D) was available in our CT system. This study defined the images with AIDR 3D reconstruction that was widely used in low-dose CT as the original image and evaluated the additional value of 4D-SF. It might have been necessary to evaluate whether 4D-SF was effective for dynamic CTP using traditional images such as filtered back-projection images. Fourth, 4D-SF could not be compared with other post-processing techniques for dynamic myocardial CTP (12, 26). However, the strength of the 4D-SF lies in its commercial availability for routine clinical use. Finally, the impact of 4D-SF on the diagnostic performance for detecting myocardial ischemia was not evaluated because we focused on the effects on radiation dose reduction in the present study. The capacity of 4D-SF might be affected by the patients' physiques and CAD severity. Multicenter prospective trials are needed to evaluate the feasibility of dynamic myocardial CTP with 4D-SF in detecting myocardial ischemia.

In conclusion, 4D-SF works in the time domain to reduce image noise in dynamic CTP data while maintaining spatial resolution. It is a promising method for improving image quality and reducing radiation dose in dynamic myocardial CTP imaging. Future studies are needed to evaluate the diagnostic performance of dynamic myocardial CTP imaging combined with ultra-low dose protocols and 4D-SF compared with established reference standards such as invasive FFR for detecting myocardial ischemia in clinical practice.

## Data Availability

The raw data supporting the conclusions of this article will be made available by the authors, without undue reservation.
